# Relationships Between Anxiety Symptoms, Hopelessness and Suicidal Ideation Among Parental Caregivers of Mandarin-Speaking Children With Speech Impairment: The Mediating Effect of Depressive Symptoms

**DOI:** 10.3389/fpsyt.2021.648885

**Published:** 2021-04-27

**Authors:** Si-Wei Ma, Sha Lai, Yan-Yan Yang, Zhongliang Zhou, Bin-Ting Yang, Gu-Zheng-Yue Zheng, Jianmin Gao, Li Lu

**Affiliations:** ^1^Key Laboratory of Shaanxi Province for Craniofacial Precision Medicine Research, College of Stomatology, Xi'an Jiaotong University, Xi'an, China; ^2^School of Public Health, Health Science Center, Xi'an Jiaotong University, Xi'an, China; ^3^Research Center of Stomatology, College of Stomatology, Xi'an Jiaotong University, Xi'an, China; ^4^School of Public Policy and Administration, Xi'an Jiaotong University, Xi'an, China; ^5^School of Medicine, Xizang Minzu University, Xianyang, China; ^6^Team IETO, Bordeaux Population Health Research Center, INSERM, Université de Bordeaux, Bordeaux, France

**Keywords:** parental caregiver, children, speech impairment, suicidal ideation, depressive symptoms, anxiety symptoms, hopelessness, China

## Abstract

**Background:** Determining the mental health status of parents who chronically care for a child with speech impairment is important for developing appropriate interventions to improve both parents' and children's health and achieve a win-win situation. Unfortunately, no study in China has explored this issue. This study investigated the differences in four aspects of mental health between maternal and paternal caregivers for the Mandarin-speaking children with speech impairment and determine whether depressive symptoms mediate the relationships between anxiety symptoms and suicidal ideation, hopelessness and suicidal ideation.

**Methods:** This cross-sectional questionnaire survey was conducted in February 2020 by sending a link to the predesigned electronic questionnaire in WeChat. Standardized assessment tools were employed. Hierarchical multiple logistic regression was conducted to examine the associations between various factors and suicidal ideation, and two separate structural equation models were performed to evaluate the mediating effects of depressive symptoms in the relationship between anxiety symptoms and suicidal ideation as well as between hopelessness and suicidal ideation.

**Results:** This study included 446 parental caregivers of Mandarin-speaking children with speech impairment. Paternal caregivers had greater score than maternal caregivers on loss of motivation (one of the subdomains of hopelessness). Somatic complications of the child (OR = 2.73, 95% CI: 1.09–6.67) and depressive symptoms (OR = 3.38, 95% CI: 1.83–6.30) were positively associated with caregivers' suicidal ideation. Having speech therapy of child (OR = 0.54, 95% CI: 0.29–0.98) was negatively correlated with caregivers' suicidal ideation. There was direct effect of depressive symptoms on suicidal ideation. Depressive symptoms play mediating roles on the relationships between anxiety symptoms (β = 0.171, *p* < 0.001) as well as between hopelessness and suicidal ideation (β = 0.187, *p* < 0.001).

**Conclusions:** Paternal and maternal caregivers of Mandarin-speaking children with speech impairment suffered from mental health problems. Preventive strategies and interventions to ameliorate parental psychological well-being, and health care policies to increase the accessibility to speech therapy care of children with speech impairment are imperative.

## Introduction

Involvement in childcare is one of the basic responsibilities of being parents; however, the role takes on an entirely different significance when a child experiences functional limitations and possible long-term dependence (1). Long-term care for children with chronic disabilities may lead to detrimental impacts on both somatic health and psychological well-being (1–3). Children with speech impairment are at risk of future academic and socioemotional difficulties, activity limitations and participation restrictions (4). Caring for a child with profound developmental delay, such as speech impairment, could place caregivers at risk of poor mental health status, including symptoms of emotional exhaustion, anxiety, depression, hopelessness, pessimism and possible psychological comorbidities (5–9). For example, one study in Germany found that the proportion of depressive disorders and anxiety disorders among mothers of children with speech-impairments is 11% and 28%, respectively (10).

Parental stress levels significantly increase with the severity level of a child's disability, due to the time-consuming process of necessary treatment and rehabilitation and the induced economic burden (11, 12). For example, one randomized controlled trial in the United Kingdom identified that poorer mental well-being of caregivers of children with autism in their mid-childhood was predicted by elevated child behavioral and emotional difficulties (13). One systematic review constructed the theoretical framework showing that both not changeable characteristics (e.g., gender, culture, economic burden and time since diagnosis) and changeable factors through intervention (e.g., self-esteem, hopelessness and self-compassion) moderated the relationship between autism stigma and caregiver's mental health (14). Furthermore, parents who experience heightened stress, anxiety, or depression during the child's diagnosis, treatment and prognosis process may interact more negatively with their child with deficits (15, 16), which can in turn affect children's recovery and development (17, 18).

Cultural factors also play roles in some parents' elevated stress and coping strategies (12, 19–21). In the traditional Chinese family structure, mothers most often act as the primary caregivers for children and thus are more likely take care of children with deficits and deal with public encounters, such as contact with health care providers (22, 23). Moreover, children's disabilities have been generally perceived as originating from their mother (24), which consequently makes maternal caregivers more vulnerable to social stigma (25). In addition to the influence of Confucian culture (24, 26), perceived stigma may generate negative self-evaluation and increase negative emotions such as anger, despair and unhappiness (27), thus leading mothers to experience additional psychological stress on top of the pressure from daily routines and adaptation and uncertainty about the prognosis of children's disease condition (28). The above depictions motivated us to explore the differences in mental health status between maternal and paternal caregivers of children with speech impairment and their potential relationships.

China has the largest population of children under five (29), and a national survey showed that the prevalence of speech disability among children aged 0–3 and 4–6 years was 12.5 and 25.2 per 10,000 children, respectively (30). Determining the mental health status of parents who chronically care for a child with speech impairment is therefore important for developing appropriate interventions to improve both parents' and children's health and achieve a win-win situation. Unfortunately, no study in China so far has explored this issue. In addition, individuals with possible depression, anxiety, poorer mental well-being etc., who take care of family members with diseases have been shown to have a higher likelihood of suicide ideation (31–33) and could eventually lead to suicide, which provides us a clue to explore underlying mechanisms of relationships between other mental health problem and suicidal ideation among this population.

Therefore, this study aimed to (1) explore the factors associated with parents' mental health problems; (2) investigate the differences in four mental health outcomes (i.e., anxiety symptoms, hopelessness, depressive symptoms and suicidal ideation) between maternal and paternal caregivers for the Chinese children with speech impairment; (3) examine the direct effects of anxiety symptoms, hopelessness and depressive symptoms on suicidal ideation; and (4) investigate whether depressive symptoms mediate the relationships between anxiety symptoms and suicidal ideation, hopelessness and suicidal ideation using structural equation modeling. According to previous studies, we hypothesized that there would be differences between maternal and paternal caregivers in mental health outcomes of interest, there would be direct effects of anxiety symptoms, hopelessness and depressive symptoms on suicidal ideation and that the relationships between anxiety symptoms and suicidal ideation, hopelessness and suicidal ideation could be mediated by depressive symptoms.

## Methods

### Study Design and Data Collection

This cross-sectional questionnaire survey was conducted in Shaanxi Province of China in February 2020. A convenient sampling method was used to recruit the participants. We included mothers and/or fathers caring for Mandarin-speaking children with cleft lip and palate (ICD-10: Q35, Q37) and other speech impairments (ICD-11: 7A10; ICD-10: F80.0) who visited the Cleft Lip and Palate Center of Stomatological Hospital of Xi'an Jiaotong University. The center provides services of diagnosis, therapy (surgical, speech and other related therapies). A link to the predesigned electronic questionnaire was sent in WeChat (a Chinese multipurpose messaging, social media and mobile payment app) groups, and electronic cash coupons were provided as incentives after completion. Caregivers voluntarily participated in this anonymous survey, and all participants provided the informed consent. Questionnaires could be processed and filled only if the participants ticked on the checkbox in the beginning page, indicating they have read and acknowledged the informed consent. We technically set the restrictions that (1) each WeChat user can participate only once and (2) questionnaires with missing answers cannot be successfully submitted, thus guaranteeing the study quality control. We followed the Strengthening the Reporting of Observational Studies in Epidemiology (STROBE) guidelines to report this study (34).

### Assessment

#### Basic Characteristics

Basic characteristics of children [i.e., age in years, type of clefts (cleft lip and palate (CLP)/cleft palate only (CPO), others with speech impairment), somatic complications (no/yes), speech therapy (no/yes)] and personal and family characteristics of caregivers [i.e., education level (high school and below/college and above), spouse's education level (high school and below/college and above), number of children in the family (one/two or more), employment status of child's mother (full time/part time/unemployed), family residence areas (urban/rural), self-perceived family economy (poor/general/rich) and housing status of the family (self-owned housing/self-owned housing (three generations living together)/rented housing)] were collected.

#### Depressive Symptoms

The validated Chinese version of the 20-item Center for Epidemiologic Studies of Depression Symptom Scale (CES-D) with satisfactory psychometric properties was used to evaluate the severity of depressive symptoms during the past week (35–37). Each item is rated on a scale ranging from 0 (rarely) to 3 (most or all of the time). The total score ranges from 0 to 60, and a score of 16 or more indicates possible depression (35, 36).

#### Anxiety Symptoms

We used the validated Chinese version of the 7-item Generalized Anxiety Disorder Scale (GAD-7) (Cronbach's α: 0.888) to assess the anxiety symptoms emerged during the last 2 weeks (38, 39). Response options for each item ranged from “not at all” (0) to “nearly every day” (3). The total score ranges from 0 to 21, and the higher scores indicate more severe clinical impairment. In our study, those who had a score of 10 or greater were considered to have moderate and severe anxiety symptoms (38).

#### Hopelessness

The caregivers' perceived level of hopelessness was evaluated using the Beck Hopelessness Scale (BHS), which has 20 true-false items (40). It was validated in Chinese population (Cronbach's α: 0.85) (41) and has been widely used (42, 43). The total score ranges from 0 to 20; a higher score reflects a greater level of hopelessness, and those with a score of 9 or greater are considered to have a moderate or severe level of hopelessness (44). The scale helps to assess three aspects of hopelessness: feelings about the future (items 1, 6, 13, 15, and 19), loss of motivation (items 2, 3, 9, 11, 12, 16, 17 and 20) and future expectations (items 4, 7, 8, 14 and 18) (40, 41). Furthermore, the predictive validity of the BHS for suicide attempts and suicide has been demonstrated (45).

#### Suicidal Ideation

We used the fourth and fifth items of the Beck Scale for Suicidal Ideation (BSS) to assess lifetime suicidal ideation (SI) (46); those who answered “yes” to at least one question would be considered as having SI. It is a widely used self-reported tool (47, 48), and the Chinese version has been validated (49).

### Statistical Analyses

We described the sociodemographic and clinical characteristics with the frequency (*n*) and percentage (%) or the mean and standard deviation (SD), as appropriate. The normality of continuous variables was tested using the Shapiro–Wilk test. The correlations between the score of depressive symptoms, anxiety symptoms and hopelessness among the included caregivers were examined using Spearman's correlation (normally distributed data) or Pearson's correlation (non-normal distributed) data. χ^2^ tests and Mann–Whitney *U*-tests were performed to examine potential statistically significant differences between paternal and maternal caregivers regarding their mental health status.

Hierarchical multiple logistic regression was conducted to examine the associations between various factors and suicidal ideation (dependent variable). In step 1, characteristics of children (age years, type of clefts, somatic complications, speech therapy) were added. In step 2, personal and family characteristics of caregivers (educational level, spouse's education level, employment status of the child's mother, number of children in the family, family residence areas, self-perceived family economy, and housing status of the family) were added. Anxiety symptoms, hopelessness and depressive symptoms (categorical variables) were added in the third step. We used the R-square change (ΔR^2^) to indicate the predictive power of each group of predictor (s) when adjustments were made for previous predictor (s). ANOVA was also conducted to compare the hierarchical models. We used odds ratios (*ORs*) and their 95% confidence intervals (*CIs*) to express the results.

Two separate structural equation models (SEMs) using the R lavaan package (50) were performed to evaluate the hypotheses of the mediating effects of depressive symptoms in the relationship between anxiety symptoms and suicidal ideation and in the relationship between hopelessness and suicidal ideation (scores were used). Basic characteristics of children and personal and family characteristics of caregivers in the third step of the hierarchical multiple logistic regressions were adjusted in the SEM. As recommended, a comparative fit index (CFI) ≥ 0.90, a root mean square error of approximation (RMSEA) <0.08, a standardized root mean square residual (SRMR) <0.08 and a Tucker-Lewis index (TLI) ≥ 0.95 indicate satisfactory fit (51, 52). Data analyses were performed using R Studio software (Version 1.2.1335, ©2009–2019 R Studio, Inc.), the significance level was set at *p* < 0.05 (two tailed).

### Ethical Approval

The study protocol was approved by the Ethics Committee of Health Science Center of Xi'an Jiaotong University (No.2019-754).

## Results

### Characteristics of the Caregivers

We analyzed data from 446 caregivers of children with speech impairment, including 338 mothers (75.8%) and 108 fathers (24.2%). Table 1 shows the basic characteristics of the children with speech impairment whose parents participated in the study, and Table 2 shows the personal and family characteristics of the caregivers. Among the participants, 53.8 and 32.3% were taking care of children with cleft palate only and cleft lip and palate, respectively, and 13.9% of children had other speech impairment. A total of 43.3% (*n* = 193) of the participants' children have received or are receiving speech therapy.

**Table 1:** Basic characteristic of the children whose parents participated in the study (*N* = 446).

**Variables**	**Categories**	***N* (%)**
Age years (Mean ± SD)		3.88 (2.91)
Sex	Male	272 (61.0)
	Female	174 (39.0)
Type of clefts	Cleft lip and palate (CLP)	144 (32.3)
	Cleft palate only (CPO)	240 (53.8)
	Others with speech impairment	62 (13.9)
Somatic complications	No	416 (93.3)
	Yes	30 (6.7)
Speech therapy	No	253 (56.7)
	Yes	193 (43.3)

*SD: standard deviation*.

**Table 2:** Personal and family characteristics of parental caregiver who participated in this study (*N* = 446).

**Variables**	**Categories**	***N* (%)**
Education level	High school and below	234 (52.5)
	College and above	212 (47.5)
Spouse's education level	High school and below	238 (53.4)
	College and above	208 (46.6)
Number of children in the family	One	226 (50.7)
	Two or more	220 (49.3)
Employment status of child's mother	Full time	158 (35.4)
	Part time	56 (12.6)
	Unemployed	232 (52.0)
Family residence areas	Urban	245 (54.9)
	Rural	201 (45.1)
Self-perceived family economy	Poor	76 (17.0)
	General	357 (80.0)
	Rich	13 (2.9)
Housing status of the family	Self-owned housing	203 (45.5)
	Self-owned housing (three generations living together)	183 (41.0)
	Rented housing	60 (13.5)
Self-perceived effects of speech therapy	No improvement	71 (15.9)
	Improved	122 (27.4)
	Missing	253 (56.7)

A total of 31.6% of the participants showed possible depression symptoms (CES-D score ≥ 16), and the proportion of participants with moderate and severe anxiety symptoms (GAD-7 score ≥ 10) was 15.2% (Table 3). A total of 59 (13.2%) caregivers perceived that they had feelings of hopelessness, and 80 (17.9%) had suicidal ideation.

**Table 3:** Mental health outcomes between paternal and maternal caregivers.

**Variables**		**Overall (*N* = 446)**	**Father (*n* = 108)**	**Mother (*n* = 338)**	p^*^
Depressive symptoms (*n*, %)	No	305 (68.4)	74 (68.5)	231 (68.3)	1.00
	Yes	141 (31.6)	34 (31.5)	107 (31.7)	
Anxiety symptoms (*n*, %)	No	378 (84.8)	93 (86.1)	285 (84.3)	0.77
	Yes	68 (15.2)	15 (13.9)	53 (15.7)	
Hopelessness (*n*, %)	No	387 (86.8)	92 (85.2)	295 (87.3)	0.69
	Yes	59 (13.2)	16 (14.8)	43 (12.7)	
Suicide ideation (*n*, %)	No	366 (82.1)	94 (87.0)	272 (80.5)	0.16
	Yes	80 (17.9)	14 (13.0)	66 (19.5%)	
Score of CESD-20	Mean (SD)	13.8 (8.6)	14.4 (9.7)	13.6 (8.2)	0.58
Score of GAD-7	Mean (SD)	4.84 (5.0)	4.83 (5.7)	4.85 (4.7)	0.33
Score of BHS-20	Mean (SD)	4.64 (3.4)	4.69 (3.5)	4.63 (3.4)	0.99
Feelings about the future	Mean (SD)	0.60 (0.9)	0.50 (0.9)	0.63 (0.9)	0.094
Loss of motivation	Mean (SD)	1.39 (1.5)	1.79 (1.7)	1.27 (1.4)	**0.004**
Future expectations	Mean (SD)	1.61 (1.5)	1.54 (1.4)	1.64 (1.5)	0.631

* *X^2^ test or Mann–Whitney U-test. Bold values: p < 0.05*.

*BHS-20: the 20-item Beck hopelessness scale; CESD-20: The 20-item Center for Epidemiologic studies of Depression Symptom Scale; GAD-7: The 7-item Generalized Anxiety Disorder Scale; SD: Standard deviation*.

Spearman's correlation analyses showed a significant correlation between the score for depressive symptoms and anxiety symptoms (*r* = 0.539, *p* < 0.001) anxiety symptoms and hopelessness (*r* = 0.381, *p* < 0.001) and depressive symptoms and hopelessness (*r* = 0.613, *p* < 0.001).

### Mental Health Status Between Paternal and Maternal Caregivers

Except for paternal caregivers who had a greater score than maternal caregivers on the domain of loss of motivation in hopelessness (1.79 ±1.7 vs. 1.27 ± 1.4; *p* = 0.004), no significant difference was found between paternal and maternal caregivers with respect to depressive and anxiety symptoms, suicidal ideation and the overall score of hopelessness (Table 3).

### Results of Hierarchical Regression Analyses

The results of hierarchical regression analyses are shown in Table 4. In total, characteristics of children and personal and family characteristics of caregivers accounted for 6.2% of the variance (Step 2). When anxiety symptoms, hopelessness and depressive symptoms were examined in step 3, we captured an additional 6.7% of variance in suicidal ideation beyond the effects of the characteristics of children and personal and family characteristics of caregivers (Δ*R*^2^ = 0.067, *F* = 28.19, *p* < 0.001), showing that somatic complications of the child (*OR* = 2.73, *95% CI*: 1.09–6.67, *p* = 0.028) and depressive symptoms (*OR* = 3.38, 95% CI: 1.83–6.30, *P* < 0.001) were positively associated with caregivers' suicidal ideation, and having speech therapy of the child (*OR* = 0.54, *95% CI*: 0.29–0.98, *p* = 0.046) was negatively correlated with caregivers' suicidal ideation.

**Table 4:** Results of hierarchical regression analyses of suicidal ideation in caregivers.

**Variables**	**Step 1**	**Step 2**	**Step 3**
	***OR (95% CI)***	***OR (95% CI)***	***OR (95% CI)***
**Characteristics of children**			
Age years	0.93 (0.82–1.04)	0.93 (0.81–1.04)	0.92 (0.80–1.04)
Sex (Female)	1.03 (0.60–1.76)	1.12 (0.64–1.97)	1.28 (0.71–2.30)
**Type of clefts of the child**			
CPO	0.96 (0.52–1.77)	1.03 (0.55–1.94)	1.02 (0.53–1.98)
Others with speech impairment	1.30 (0.54–2.98)	1.80 (0.71–4.46)	1.92 (0.73–4.90)
Somatic complications (Yes)	2.44 (1.04–5.48) ^*^	2.50 (1.04–5.81)^*^	2.73 (1.09–6.67)^*^
Speech therapy (Yes)	0.51 (0.28–0.89) ^*^	0.51 (0.28–0.90)^*^	0.54 (0.29–0.98)^*^
**Personal and family characteristics of caregivers**			
Education level (College and above)		0.81 (0.38–1.72)	0.73 (0.32–1.64)
Spouse's education level (College and above)		1.05 (0.50–2.20)	1.25 (0.57–2.78)
**Employment status of child's mother**			
Part time		1.46 (0.60–3.37)	1.81 (0.73–4.36)
Unemployed		1.28 (0.67–2.47)	1.47 (0.75–2.96)
Number of children in the family (Two or more)		0.84 (0.49–1.44)	0.93 (0.53–1.65)
Family residence areas (Rural)		0.95 (0.51–1.76)	0.78 (0.40–1.49)
**Self–perceived family economy**			
General		0.72 (0.36–1.47)	0.99 (0.47–2.16)
Rich		0.32 (0.02–2.10)	0.58 (0.03–4.01)
**Housing status of the family**			
Self–owned (three generations living together)		1.44 (0.79–2.66)	1.49 (0.79–2.81)
Rented housing		2.24 (1.04–4.75)^*^	2.17 (0.96–4.85)
**Anxiety symptoms** (Yes)			0.88 (0.41–1.81)
**Hopelessness** (Yes)			1.77 (0.86–3.59)
**Depressive symptoms** (Yes)			3.38 (1.83–6.30)^***^
***R**^**2**^*	0.039	0.062	0.130
***ΔR**^**2**^*		0.024	0.067
***F (p)***		9.99 (0.441)	28.19 (<0.001)

*CPO, cleft palate only; SI, suicidal ideation*.

*Step 1: adjusted for Characteristics of children*.

*Step 2: Model 1 variables + personal and family characteristics of caregiver*.

*Step 3: Model 2 variables + anxiety symptoms + hopelessness + depressive symptoms*.

* *p < 0.05*,

*** *p < 0.001 (two-tailed)*.

### Results of Structural Equation Modeling

**Figure 1:**
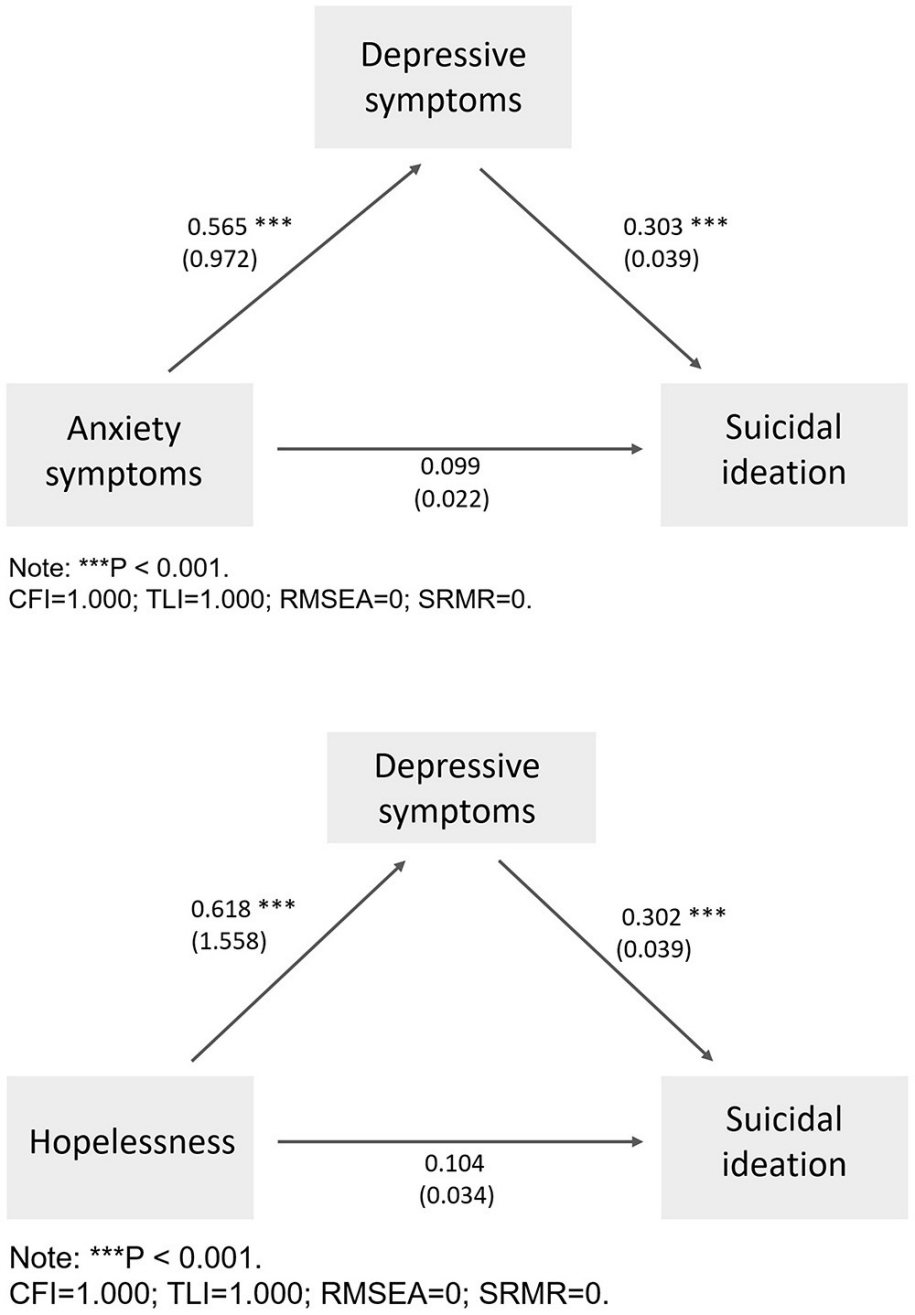
Final model with the standardized coefficients (β), and unstandardized coefficients (Beta) presented in the parentheses. (*N* = 446).

Figure 1 show the results of structural equation modeling. In both models, there were direct effects of depressive symptoms on suicidal ideation (model1: β = 0.303, *p* < 0.001; model 2: β = 0.302, *p* < 0.001). Anxiety symptoms and hopelessness did not have significant effects on suicidal ideation (*p* > 0.05). The SEMs revealed the mediating effects of depressive symptoms on the association between anxiety symptoms and suicidal ideation (β = 0.171, *p* < 0.001) and the relationship between hopelessness and suicidal ideation (β = 0.187, *p* < 0.001). Goodness-of-fit indices (CFI = 1.000; TLI = 1.000; RMSEA = 0; SRMR = 0 for both models) indicated a satisfactory fit among the two SEMs.

## Discussion

This study is the first to examine the mental well-being of parental caregivers of Chinese Mandarin-speaking children with speech impairment, in which 86.1% were caregivers of children with cleft palate. Our findings provide considerable insights into psychological amelioration in the population of interest and offer clues for both clinical practice and future research.

No difference was found in the mental health outcomes of interest between maternal and paternal caregivers; the only exception is that father caregivers had greater scores than mothers on loss of motivation, one subdomain of hopelessness. Unlike our findings, one study in the USA comprised 644 mothers and 519 fathers of children with cleft lip and/or palate suggested that mothers scored less favorably on anxiety and depressive symptoms and stress than fathers (53). Another study focused on parents of children with intellectual disabilities identified that mothers had higher levels of daily parenting stress than fathers (54). The discrepancies may be attributed to the divergences of many aspects, such as social culture and the assessment tools used. For example, the study in the USA used the Hospital Anxiety and Depression Scale (53), while we employed CES-D scale and GAD-7 to assess depressive and anxiety symptoms. There is very little research on parents' loss of motivation. Individualist theories predicted that, for single fathers, they are short of both the expressive skills and the internal motivation to provide the intense intimacy necessary for nurturing young children (55), but the marital status was not investigated in our study. On the contrary, another study involved 103 parents of children with hearing loss in South Africa found that, fathers scored higher on parental warmth, confidence, positive interaction, communication and satisfaction than mothers (56). In our study, only approximately 25% of sample were fathers (108/446). As previously recorded, the husband-father's motivation to share parenting responsibilities can bring benefit to the marital relationship and also the child's development (57). However, the roles of fathers were usually neglected and there was a preference for focusing mainly on mothers' psychological well-being and excluding fathers in most studies concerning the experiences of parents caring for a child with a cleft (58), which should be further taken into consideration.

As far as we know, suicidal ideation among parental caregivers of children with speech impairment has barely been reported, especially in Chinese studies. The prevalence of suicidal ideation (17.9%) among our sample was <12.2% in one study on carers of children (aged 4–13 years) affected by HIV (59). Previous study in Oman found that 45.9 and 48.6% of the parents caring for children with autism spectrum disorder having anxiety and depression symptoms, respectively (60), which are greater than our figures. Compared with our data, another study in Germany among mothers of children with speech-impairments reported lower prevalence of depressive disorders (11%) and higher anxiety disorders (28%) (10). While the data as regards to the prevalence of hopelessness were hardly ever clearly reported.

Using hierarchical regression analyses, we further determined that parental caregivers with depressive symptoms and parents whose child has somatic complications were approximately 2.7 and 3.4 times more likely to have suicidal ideation, respectively. It was documented that stress levels of parents greatly escalated with the severity level of the child's disability (e.g., autism, Down's syndrome, cerebral palsy) (12). Somatic complications typically act as a marker for severity of some disease (61), the former thus was speculated to increase parents' stress levels, which was associated with increased risk of suicidal behaviors (62). Hierarchical regression analyses also revealed that speech therapy for children was a protective factor against suicidal ideation. Among parental caregivers whose child received speech therapy, 63.3% (122/193) of the caregivers in our study perceived the therapy as effective. Under most circumstances, therapy interventions are beneficial for both caregivers and patients, and children receiving therapies are able to bring comfort to their parents. Unfortunately, only 43.3% of respondents reported speech therapy of their children. There are limited specialized hospitals being able to provide speech therapy, and a shortage of at least 100,000 Speech-language pathologists (SLPs) was estimated in China (63). Those chose to forego speech therapy (56.7%) possibly because of the financial burden and poor accessibility of treatment and rehabilitative services. Health policy-makers should consider the issue and implement effective approaches, such as training programs and allocations of SLPs and consideration of including speech therapy in health insurance, to benefit the whole family with a child suffering from speech impairment and needs speech therapy.

Hopelessness, anxiety and depression were conceptualized as risk factors for suicidal ideation in many existing studies (64–66). Unexpectedly, no direct relationships were found between hopelessness, anxiety and suicidal ideation in either regression analyses or structural equation models on our targeted population, which was not consistent with study hypothesis. However, we do find a direct relationship between depressive symptoms and suicidal ideation. Similarly, one previous study analogous the targeted population with ours, which was on caregivers of individuals with disabilities, and elucidated that caregivers with probable depression had greater suicidal ideation (33). There were indirect effects from anxiety symptoms and hopelessness on suicidal ideation that were mediated by depressive symptoms. A prior study introduced the bidirectional relationship between anxiety and depression in the general population (67), and the links between hopelessness, anxiety and depression were reported among the parents of children with cancer (68), which helped us to explain the mediating role of depressive symptoms. However, further studies should retest the potential mechanisms.

In our study, 86% of the participants' children had cleft lip and/or palate, which is usually diagnosed at birth. It is challenging to care for a child with congenital malformation at birth and sequence therapy until the adult period. Our study extend the existing literature, especially in China, by indicating the poor mental health status of parental caregivers of Mandarin-speaking children with speech impairment, and associated factors to the worst case, i.e., suicidal ideation, as well as the mechanisms of relationships with other mental health indicators. Interventions and preventive strategies to ameliorate their psychological well-being are warranted. For example, stress management interventions could be adopted since they were determined to effectively reduce the reported stress of parents caring for children with developmental disabilities (69).

Our study is the first to take advantage of SEM to best identify the underlying mechanisms of psychological factors that contribute to suicidal ideation among maternal and paternal caregivers of Mandarin-speaking children with speech impairment. However, several limitations should be noted. First, the properties of cross-sectional design could partly reduce the value of SEM and it is impossible to test the causal relationship. Second, we recruited participants from one city in China, which limited the generalizability of the findings to other areas or countries. Third, only approximately one-fourth of the sample were fathers, since we followed the principle of voluntary, those took part in this study might be partly different from those did not, especially those who may present themselves in the hospital and who did not. Plus the fact that there is an imbalance in sample size between paternal and maternal caregivers, which could lead to potential selection bias. Besides, either father or mother of one child with cleft lip and/or palate and others with speech impairment, or both parents (very few) of one child could have taken part in this anonymous survey. It is therefore unable to process the matching procedure and compare outcomes of interest on paired parents, which calls for future paired sample studies. Lastly, other mental health outcomes of parental caregivers except what we have investigated should be further studied, as the mechanisms behind the relationships among mental health outcomes are quite complex and other variables could also play a role.

In conclusion, maternal and paternal caregivers of Mandarin-speaking children with speech impairment suffered from mental health problems, and fathers were more likely to lose motivation. Depressive symptoms played a mediating role in the relationships between anxiety symptoms and suicidal ideation, hopelessness and suicidal ideation. Our findings provide insights into the imperative initiation and implementation of clinical- and community-based preventive strategies and interventions to ameliorate parental psychological well-being. Health policy-makers should moreover consider and develop effective approaches to increase the accessibility to speech therapy care of children with speech impairment to benefit the whole family.

## Data Availability Statement

The raw data supporting the conclusions of this article will be made available by the authors, without undue reservation.

## Ethics Statement

The studies involving human participants were reviewed and approved by the Ethics Committee of Health Science Center of Xi'an Jiaotong University. The patients/participants provided their written informed consent to participate in this study.

## Author Contributions

S-WM, LL, JG, and ZZ designed the study. S-WM, Y-YY, and B-TY conducted the data collection. LL, G-Z-YZ, and SL analyzed and interpreted the data. S-WM and LL drafted the manuscript. S-WM, LL, and SL critically revised the manuscript. All the authors approved the final version of this article for publication.

## Conflict of Interest

The authors declare that the research was conducted in the absence of any commercial or financial relationships that could be construed as a potential conflict of interest.

## References

[1] Raina P O'Donnell M Rosenbaum P Brehaut J Walter SD Russell D. The health and well-being of caregivers of children with cerebral palsy. Pediatrics. (2005) 115:e626–36. 10.1542/peds.2004-1689 15930188

[2] King G King S Rosenbaum P Goffin R. Family-centered caregiving and well-being of parents of children with disabilities: linking process with outcome. J Pediatr Psychol. (1999) 24:41–53. 10.1093/jpepsy/24.1.41

[3] Gowda MR Pai NB Vella SLC. A pilot study of mental health and quality-of-life of caregivers of children with cleft lip/palate in India. Ind J Psychiatry. (2013) 55:167–9. 10.4103/0019-5545.111456 23825852 PMC3696241

[4] McCormack J McLeod S McAllister L Harrison LJ. A systematic review of the association between childhood speech impairment and participation across the lifespan. Int J Speech Lang Pathol. (2009) 11:155–70. 10.1080/17549500802676859

[5] Fisman S Wolf L Ellison D Freeman T. A longitudinal study of siblings of children with chronic disabilities. Can J Psychiatry Rev Can Psychiatr. (2000) 45:369–75. 10.1177/070674370004500406 10813071

[6] Chan JB Sigafoos J. Does respite care reduce parental stress in families with developmentally disabled children? Child Youth Care Forum. (2001) 30:253–63. 10.1023/A:1014467226528

[7] Pruchno RA Meeks S. Health-related stress, affect, and depressive symptoms experienced by caregiving mothers of adults with a developmental disability. Psychol Aging. (2004) 19:394–401. 10.1037/0882-7974.19.3.394 15382990

[8] Vinayak S Dhanoa SK Vinayak R. Relationship of hopelessness, depression and quality of life in mothers of persons with disabilities. Int J Innov Appl Stud. (2016) 17:306.

[9] Sloan CJ Mailick MR Hong J Ha JH Greenberg JS Almeida DM. Longitudinal changes in well-being of parents of individuals with developmental or mental health problems. Soc Sci Med. (2020) 264:113309. 10.1016/j.socscimed.2020.113309 32858491 PMC7441882

[10] Wink M Rosanowski F Hoppe U Eysholdt U Grässel E. Subjective burden in mothers of speech-impaired children. Folia Phoniatr Logop. (2007) 59:268–72. 10.1159/000104465 17726330

[11] Rudolph M Rosanowski F Eysholdt U Kummer P. Anxiety and depression in mothers of speech impaired children. Int J Pediatr Otorhinolaryngol. (2003) 67:1337–41. 10.1016/j.ijporl.2003.08.042 14643478

[12] DeLambo D Chung W Huang WH. Stress and age: a comparison of asian american and non-asian american parents of children with developmental disabilities. J Dev Physical Dis. (2011) 23:129–41. 10.1007/s10882-010-9211-3

[13] Salomone E Leadbitter K Aldred C Barrett B Byford S Charman T. The association between child and family characteristics and the mental health and wellbeing of caregivers of children with autism in mid-childhood. J Autism Dev Disord. (2018) 48:1189–98. 10.1007/s10803-017-3392-x 29177606 PMC5861164

[14] Papadopoulos C Lodder A Constantinou G Randhawa G. Systematic review of the relationship between autism stigma and informal caregiver mental health. J Autism Dev Disord. (2019) 49:1665–85. 10.1007/s10803-018-3835-z 30569408 PMC6450836

[15] Krueckeberg SM Kapp-Simon KA. Effect of parental factors on social skills of preschool children with craniofacial anomalies. Cleft Palate Craniof J. (1993) 30:490–6. 10.1597/1545-1569_1993_030_0490_eopfos_2.3.co_2 8218313

[16] Pope AW Tillman K Snyder HT. Parenting stress in infancy and psychosocial adjustment in toddlerhood: a longitudinal study of children with craniofacial anomalies. Cleft Palate Craniof J. (2005) 42:556–9. 10.1597/04-066r.1 16149839

[17] Dykens EM Fisher MH Taylor JL Lambert W Miodrag N. Reducing distress in mothers of children with autism and other disabilities: a randomized trial. Pediatrics. (2014) 134:e454–63. 10.1542/peds.2013-3164 25049350 PMC4187227

[18] Wei QW Zhang CH Zhang JX Luo SS Wang X. Caregiver's depressive symptoms and young children's socioemotional development delays: a cross-sectional study in poor rural areas of China. Infant Mental Health J. (2018) 39:209–19. 10.1002/imhj.21699 29485680

[19] Ryan AS Smith MJ. Parental reactions to developmental disabilities in chinese American families. Child Adol Soc Work J. (1989) 6:283–99. 10.1007/BF00755222

[20] Choi KH Wynne ME. Providing services to asian Americans with developmental disabilities and their families: mainstream service providers' perspective. Comm Mental Health J. (2000) 36:589–95. 10.1023/A:1001934202450 11079186

[21] Kwok S Wong D. Mental health of parents with young children in Hong Kong: the roles of parenting stress and parenting self-efficacy. Child Family Soc Work. (2000) 5:57–65. 10.1046/j.1365-2206.2000.00138.x

[22] Gray DE. 'Everybody just freezes. Everybody is just embarrassed': felt and enacted stigma among parents of children with high functioning autism. Soc Health Illness. (2002) 24:734–49. 10.1111/1467-9566.00316

[23] Kelso T French D Fernandez M. Stress and coping in primary caregivers of children with a disability: a qualitative study using the lazarus and folkman process model of coping. J Res Spec Educ Needs. (2005) 5:3–10. 10.1111/j.1471-3802.2005.00033.x

[24] Holroyd EE. Chinese cultural influences on parental caregiving obligations toward children with disabilities. Qual Health Res. (2003) 13:4–19. 10.1177/1049732302239408 12564260

[25] Khamis V. Psychological distress among parents of children with mental retardation in the united arab emirates. Soc Sci Med (1982). (2007) 64:850–57. 10.1016/j.socscimed.2006.10.022 17129651

[26] Haugh M Hinze C. A metalinguistic approach to deconstructing the concepts of 'face' and 'politeness' in chinese, english and Japanese. J Prag. (2003) 35:1581–611. 10.1016/S0378-2166(03)00049-3

[27] Corrigan PW Watson AC. The paradox of self-stigma and mental illness. Clin Psychol Sci Practice. (2006) 9:35–53. 10.1093/clipsy.9.1.35

[28] Mak WW Cheung RY. Psychological distress and subjective burden of caregivers of people with mental illness: the role of affiliate stigma and face concern. Comm Mental Health J. (2012) 48:270–4. 10.1007/s10597-011-9422-9 21681460

[29] Shao J. Early child development: a challenge in China. World J Pediatr. (2019) 15:6–8. 10.1007/s12519-018-0216-9 30632075

[30] Yun C Wang Z He P Guo C Chen G Zheng X. Prevalence and parental risk factors for speech disability associated with cleft palate in chinese children-a national survey. Int J Environ Res Public Health. (2016) 13:1168. 10.3390/ijerph13111168 27886104 PMC5129378

[31] O'Dwyer S Moyle W van Wyk S. Suicidal ideation and resilience in family carers of people with dementia: a pilot qualitative study. Aging Ment Health. (2013) 17:753–60. 10.1080/13607863.2013.789001 23611756

[32] Park B Kim SY Shin JY Sanson-Fisher RW Shin DW Cho J. Suicidal ideation and suicide attempts in anxious or depressed family caregivers of patients with cancer: a nationwide survey in Korea. PLoS ONE. (2013) 8:e60230. 10.1371/journal.pone.0060230 23565204 PMC3615012

[33] Huang YC Hsu ST Hung CF Wang LJ Chong MY. Mental health of caregivers of individuals with disabilities: relation to suicidal ideation. Comp Psychiatry. (2018) 81:22–7. 10.1016/j.comppsych.2017.11.003 29195106

[34] Von Elm E Altman DG Egger M Pocock SJ Gøtzsche PC Vandenbroucke JP. The strengthening the reporting of observational studies in epidemiology (STROBE) statement: guidelines for reporting observational studies. Ann Intern Med. (2007) 147:573–7. 10.7326/0003-4819-147-8-200710160-00010 17938396

[35] Radloff LS. The CES-D scale: a self-report depression scale for research in the general population. Appl Psychol Meas. (1977) 1:385–401. 10.1177/014662167700100306

[36] Rankin SH Galbraith ME Johnson S. Reliability and validity data for a Chinese translation of the center for epidemiological studies-depression. Psychol Rep. (1993) 73(3 Pt 2):1291–8. 10.2466/pr0.1993.73.3f.1291 8115582

[37] Ling Y Wei Y. Factorial structure of the CES-D scale among chinese high school students. Int J Psychol. (1993) 43:3–4.

[38] Spitzer RL Kroenke K Williams JB Löwe B. A brief measure for assessing generalized anxiety disorder: the GAD-7. Arch Intern Med. (2006) 166:1092–7. 10.1001/archinte.166.10.1092 16717171

[39] Tong X An D McGonigal A Park SP Zhou D. Validation of the generalized anxiety disorder-7 (GAD-7) among chinese people with epilepsy. Epilepsy Res. (2016) 120:31–6. 10.1016/j.eplepsyres.2015.11.019 26709880

[40] Beck AT Weissman A Lester D Trexler L. The measurement of pessimism: the hopelessness scale. J Cons Clin Psychol. (1974) 42:861. 10.1037/h0037562 4436473

[41] Kong YY Zhang J Jia SH Zhou L. Reliability and validity of the beck hopelessness scale for adolescent (In Chinese). Chin Men Health J. (2007) 21:686–9.

[42] Zhang J Lamis DA Yuanyuan K. Measuring chinese psychological traits and social support with Western developed instruments in psychological autopsy studies. J Clin Psychol. (2012) 68:1313–21. 10.1002/jclp.21907 22899280

[43] Han Y Yuan J Luo Z Zhao J Wu J Liu R. Determinants of hopelessness and depression among chinese hospitalized esophageal cancer patients and their family caregivers. Psychooncology. (2013) 22:2529–36. 10.1002/pon.3315 23703786

[44] Beck AT Steer RA. Manual for the Beck Hopelessness Scale. San Antonio, TX: Psychological Corporation (1988).

[45] Brown GK. A Review of Suicide Assessment Measures for Intervention Research With Adults and Older Adults. Philadelphia, PA: GK Brown (2001).

[46] Beck AT Kovacs M Weissman A. Assessment of suicidal intention: the scale for suicide ideation. J Cons Clin Psychol. (1979) 47:343. 10.1037/0022-006X.47.2.343 469082

[47] van Spijker BA van Straten A Kerkhof AJ. The effectiveness of a web-based self-help intervention to reduce suicidal thoughts: a randomized controlled trial. Trials. (2010) 11:1–7. 10.1186/1745-6215-11-25 20214777 PMC2841163

[48] Yang L Liu X Chen W Li L. A test of the three-step theory of suicide among chinese people: a study based on the ideation-to-action framework. Arch Suicide Res. (2019) 23:648–61. 10.1080/13811118.2018.1497563 30024342

[49] Li XY Phillips MR Tong YS Li KJ Zhang YL Zhang YP. Reliability and validity of the chinese version of beck suicide ideation scale (BSI-CV) in adult community residents. Chin Mental Health J. (2010) 24:250–5.

[50] Rosseel Y. Lavaan: an R package for structural equation modeling and more. Version 0.5–12 (BETA). J Stat Software. (2012) 48:1–36. 10.18637/jss.v048.i02

[51] Hooper D Coughlan J Mullen MR. Structural equation modelling: guidelines for determining model fit. Electro J Bus Res Meth. (2008) 6:53–60. 10.21427/D7CF7R

[52] Kline RB. Principles and Practice of Structural Equation Modeling. New York, NY: Guilford Publications (2015).

[53] Stock NM Costa B White P Rumsey N. Risk and protective factors for psychological distress in families following a diagnosis of cleft lip and/or palate. Cleft Palate Craniofac J. (2020) 57:88–98. 10.1177/1055665619862457 31378083

[54] Gerstein ED Crnic KA Blacher J Baker BL. Resilience and the course of daily parenting stress in families of young children with intellectual disabilities. J Int Dis Res. (2009) 53:981–97. 10.1111/j.1365-2788.2009.01220.x 19874449 PMC2796238

[55] Risman BJ. Can men “mother”? Life as a single father. Family Relat. (1986) 95–102. 10.2307/584288

[56] Davids RS Roman NV Schenck R. Parenting approaches of hearing mothers and fathers to children with hearing loss. Tydskrif Vir Geesteswetenskappe. (2020) 60:727–44. 10.17159/2224-7912/2020/v60n3a10

[57] Biller HB. Fathers and Families: Paternal Factors in Child Development. ABC-CLIO (1993).

[58] Nelson P Glenny AM Kirk S Caress AL. Parents' experiences of caring for a child with a cleft lip and/or palate: a review of the literature. Child Care Health Dev. (2012) 38:6–20. 10.1111/j.1365-2214.2011.01244.x 21623872

[59] Skeen S Tomlinsarahon M Macedo A Croome N Sherr L. Mental health of carers of children affected by HIV attending community-based programmes in South Africa and Malawi. AIDS Care. (2014) 26(Suppl. 1):S11–20. 10.1080/09540121.2014.906559 24766642 PMC4554389

[60] Al-Farsi OA Al-Farsi YM Al-Sharbati MM Al-Adawi S. Stress, anxiety, and depression among parents of children with autism spectrum disorder in Oman: a case-control study. Neuropsychiatr Dis Treat. (2016) 12:1943–51. 10.2147/NDT.S107103 27536117 PMC4977076

[61] Bohman H Jonsson U Von Knorring AL Von Knorring L Päären A Olsson G. Somatic symptoms as a marker for severity in adolescent depression. Acta Paediatr. (2010) 99:1724–30. 10.1111/j.1651-2227.2010.01906.x 20545935

[62] Feskanich D Hastrup JL Marshall JR Colditz GA Stampfer MJ Willett WC. Stress and suicide in the nurses' health study. J Epidemiol Commun Health. (2002) 56:95–98. 10.1136/jech.56.2.95 11812806 PMC1732078

[63] Shan C. Seize the opportunity to realize a new great development in speech-language pathology in China (In Chinese). Chinese J. Rehabil. (2016) 31:403–4.

[64] Thibodeau MA Welch PG Sareen J Asmundson GJ. Anxiety disorders are independently associated with suicide ideation and attempts: propensity score matching in two epidemiological samples. Depress Anxiety. (2013) 30:947–54. 10.1002/da.22203 24108489

[65] Qiu T Klonsky ED Klein DN. Hopelessness predicts suicide ideation but not attempts: A 10-Year longitudinal study. Suicide Life Thre Behav. (2017) 47: 718–22. 10.1111/sltb.12328 28150463 PMC5540846

[66] Ribeiro JD Huang X Fox KR Franklin JC. Depression and hopelessness as risk factors for suicide ideation, attempts and death: meta-analysis of longitudinal studies. Br J Psychiatry. (2018) 212:279–86. 10.1192/bjp.2018.27 29587888

[67] Jansson-Fröjmark M Lindblom K. A bidirectional relationship between anxiety and depression, and insomnia? A prospective study in the general population. J Psychosom Res. (2008) 64:443–9. 10.1016/j.jpsychores.2007.10.016 18374745

[68] Bayat M Erdem E Gül Kuzucu E. Depression, anxiety, hopelessness, and social support levels of the parents of children with cancer. J Pediatr Oncol Nurs. (2008) 25:247–53. 10.1177/1043454208321139 18648089

[69] Lindo EJ Kliemann KR Combes BH Frank J. Managing stress levels of parents of children with developmental disabilities: a meta-analytic review of interventions. Family Rel. (2016) 65:207–24. 10.1111/fare.12185

